# Strength of Footing with Punching Shear Preventers

**DOI:** 10.1155/2014/474728

**Published:** 2014-10-21

**Authors:** Sang-Sup Lee, Jiho Moon, Keum-Sung Park, Kyu-Woong Bae

**Affiliations:** ^1^Advanced Building Research Division, Korea Institute of Civil Engineering and Building Technology (KICT), Goyang-si, Gyeonggi-do 411-712, Republic of Korea; ^2^New Transportation Research Center, Korea Railroad Research Institute (KRRI), Uiwang-si, Gyeonggi-do 437-757, Republic of Korea

## Abstract

The punching shear failure often governs the strength of the footing-to-column connection. The punching shear failure is an undesirable failure mode, since it results in a brittle failure of the footing. In this study, a new method to increase the strength and ductility of the footing was proposed by inserting the punching shear preventers (PSPs) into the footing. The validation and effectiveness of PSP were verified through a series of experimental studies. The nonlinear finite element analysis was then performed to demonstrate the failure mechanism of the footing with PSPs in depth and to investigate the key parameters that affect the behavior of the footing with PSPs. Finally, the design recommendations for the footing with PSPs were suggested.

## 1. Introduction

The punching shear failure of the slab-to-column or the footing-to-column connection is undesirable, since it results in a brittle and catastrophic failure of the concrete structures. Extensive studies have been conducted for punching shear failure of the normal flat slab in the past decades, and large numbers of experimental databases were constructed by previous researchers [1–3]. Several methods to enhance the punching shear strength of the slab-to-column connection have been studied. Fernández Ruiz et al. [4] and Clément et al. [5, 6] studied the strengthening method of the slab-to-column connection by using prestressing technic. Pilakoutas and Li [7] developed the shearband system which is a shear reinforcement system using steel strips with high ductility. A shearhead system was developed by Corley and Hawkins [8]. Adding a steel plate to the flat slab to increase the effective column head area is another way of increasing the punching shear strength of the slab-to-column connection. As an example, a NUUL system was developed by Subedi and Baglin [9]. This NUUL system is composed of a steel plate and several U bars. The effect of fiber reinforced concrete on the punching shear failure of the slab-to-column connection has been studied by McHarg et al. [10], Cheng and Parra-Montesinos [11], Harajli et al. [12], and Nguyen-Minh et al. [13]. Also, carbon fiber reinforced polymer (CFRP) sheets [14] and funnel-shaped punching shear preventer (or UFO punching preventer) [15] have been proposed to strengthen the slab-to-column connection. Above this, some strengthening methods of existing slab-to-column connections and their basic mechanism are summarized by Koppitz et al. [16]. While numerous studied have been performed for the punching shear failure of the slab-to-column connection, the study on the punching shear failure of footing-to-column connection is still limited [17–19]. As a result, the punching shear design of footing is mainly based on the research results of the slab-to-column connection [19], and most design codes, such as CEB-FIP [20] and ACI [21], do not distinguish between the slab and footing in the design specification [19].

**Figure 1:**
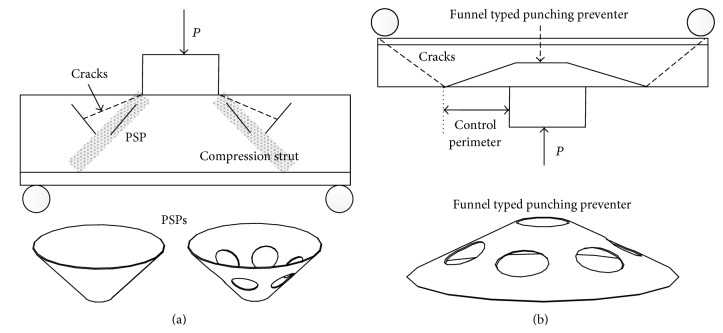
Schematic view of (a) footing with PSPs and (b) flat slab with funnel typed punching shear preventer.

This study focused on punching shear behavior of the footing-to-column connection. A new method to increase the punching shear strength and ductility of the footing-to-column connection was proposed. For the flat slab, funnel-shaped punching shear preventer shown in Figure 1(a) is sometimes used to increase the punching shear strength of flat slab-to-column connection [15]. Generally, conventional design codes [20, 21] define the punching shear strength in terms of a nominal shear capacity on a control perimeter at a certain distance from the column perimeter. When the funnel-shaped punching shear preventer shown in Figure 1(a) is used for the flat slab, control perimeter can be determined by the size of the funnel-shaped punching shear preventer. Thus, increasing the size of the funnel-shaped punching shear preventer can produce higher punching shear strength. However, funnel-shaped punching shear preventer shown in Figure 1(a) is not suitable for large scale construction, such as high rise building column-to-footing or bridge pier-to-footing connection, since the size of the funnel-shaped punching shear preventer becomes very large, which makes it hard to handle.

As an alternative, in this study, four small punching shear preventers (PSPs) are inserted into the footing, as shown in Figure 1(b). PSP has cone shape and it is made of steel. Basically, PSP has smooth surface. However, the bond strength between PSP and concrete can be improved by introducing punched holes, as shown in Figure 1(b). By inserting PSPs into the footing, the following benefits can be expected: (1) propagation of shear crack could be effectively prevented; (2) PSP could enhance the compression strut developed by the axial compression from the column. Therefore, enhanced punching shear strength and ductile failure of the footing-to-column connection are expected by using the PSPs.

In this study, the validation and effectiveness of the PSP were verified through a series of tests and nonlinear finite element analysis. A total number of five large scale test specimens were constructed and tested in order to investigate the effect of PSP on the punching shear strength and the failure mode of the footing with PSP. Then, a series of parametric studies was conducted to demonstrate the failure mechanism in depth and to study the effect of key parameters of PSP on the behavior of the footing. Finally, the design recommendations for the footing with PSPs were suggested.

## 2. Experimental Study

### 2.1. Description of Test Specimen

**Figure 2:**
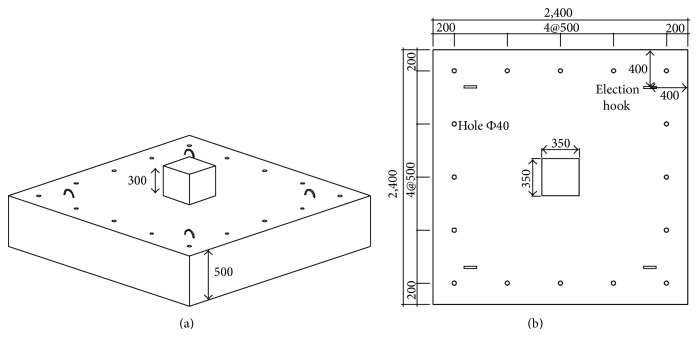
Dimensions of the test specimen.

A total of five large scale test specimens were constructed and tested. All five footings had the same dimensions and reinforcing bar layout.  Figure 2 shows the dimensions of the footing. The footing had a square shape with side length of 2.400 mm and depth of 500 mm. The axial load was applied through the square column in the center of the footing, where the width and the height of the column were 350 mm and 300 mm, respectively. 40 mm diameter holes were spaced at 500 mm to insert the anchor bars, as shown in Figure 2.

**Figure 3:**
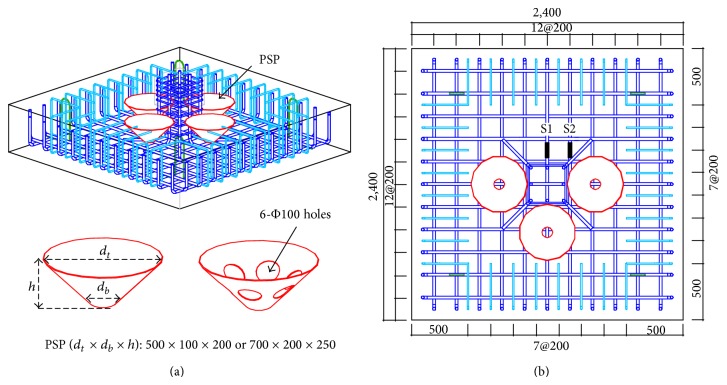
Reinforcement and PSP layout for the test specimen.

Figure 3 shows the layout of the reinforcing bars and PSPs. The bottom flexural reinforcing bars consisted of SD500 D25 bars spaced at 200 mm, where the yield stress and the diameter of SD500 D25 bar are 530 MPa and 25.4 mm, respectively. The resulting flexural reinforcement ratio was approximately 0.5%. The top flexural reinforcing bars spaced at 200 mm were installed with the length of 500 mm at the edge of the test specimens, as shown in Figure 3, to prevent the premature cracking and failure due to negative bending moment at the edges. The SD500 D25 bars were also used for the top reinforcing bars. According to ACI code [21], concrete cover for concrete casted against and permanently exposed to earth is 3 in (76.2 mm). Thus, 80 mm of concrete cover depth was adopted for the top and the bottom reinforcing bars. To prevent the premature failure of the column during the test, the column was reinforced by using eight SD500 D25 longitudinal bars and SD500 D13 stirrups spaced at 50 mm, where the yield stress and the diameter of SD500 D13 bar are 530 MPa and 12.7 mm, respectively. As shown in Figure 3, two strain gauges (S1 and S2) were installed at the bottom flexural reinforcing bars to measure the longitudinal strain of the bars.

All test specimens were casted at the same time. Type I ordinary Portland cement was used with water-to-cement ratio of 0.48. Crushed shape coarse aggregate was used where the maximum size was 25 mm. From the material test, the average compressive strength was 25.2 MPa. PSPs were made of steel where yield stress was 400 MPa from mill sheet.

The names and the test parameters for each specimen are shown in Table 1. P0_RC is the reference test specimen, where PSPs were not installed in the footing. For P5_3.2, P5_6, P5H_3.2, and P7_3.2 specimens, four PSPs were inserted into the footing, as shown in Figure 3. Two different sizes of PSPs were used for the test (500 × 100 × 200 and 700 × 200 × 250), as shown in Table 1, where *d*_*t*_ and *d*_*b*_ represent the diameters of the top and bottom of PSP, respectively. *h* is the height of PSP. For both PSP (500 × 100 × 200) and PSP (700 × 200 × 250), PSP was inclined with 45° angle. PSP (500 × 100 × 200) was used for P5_3.2, P5_6, and P5H_3.2 specimens, while PSP (700 × 200 × 250) was applied to P7_3.2 specimen. The thickness of the PSP was 3.2 mm except P5_6 specimen. The thickness of PSP used for P5_6 specimen was 6 mm. In the case of P5H_3.2 specimens, six punched holes were uniformly distributed along the centerline of PSP where the diameter of the holes was 100 mm.

From Table 1, it can be known that the effect of PSP can be investigated by comparing the results of P0_RC specimen with other test results. Also, the effect of thickness, size, and holes of PSP can be evaluated by comparing the test results of P5_3.2 specimen with those of P5_6, P7_3.2, and P5H_3.2 specimens, respectively.

**Figure 4:**
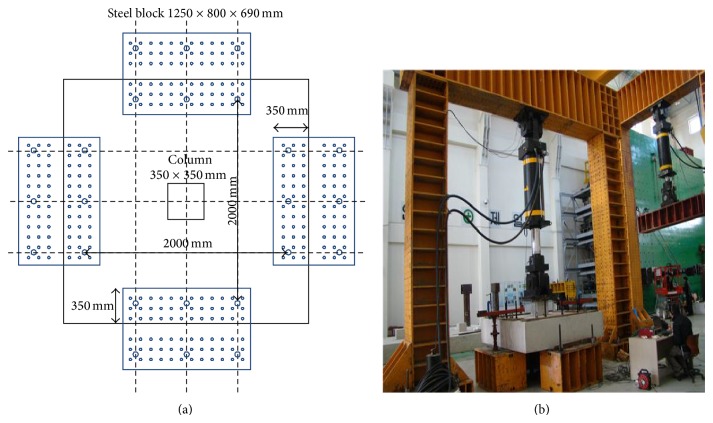
Test setup.

Figure 4 shows the test setup used in this study. The test specimen was placed on the reaction block and anchor bars were inserted to center the specimen. It should be noted that the anchor bars were not fastened. Thus, it allows upward vertical movement of the test specimen at the position of anchor bar. The vertical displacement was applied by using the actuator shown in Figure 4. The vertical displacement of the footing was measured by the linear variable differential transducers (LVDTs) installed below the bottom of the test specimen.

### 2.2. Discussion of Test Results

**Figure 5:**
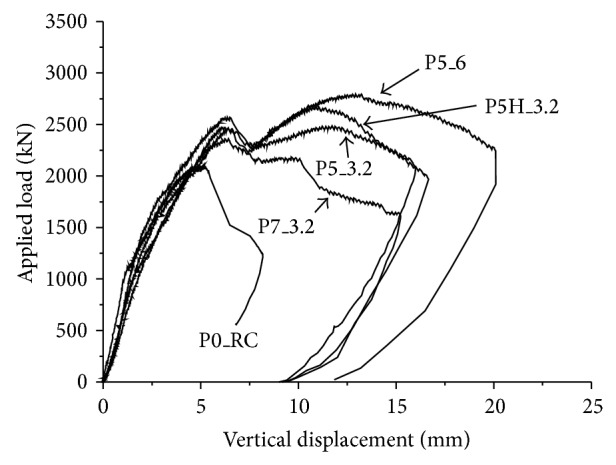
Applied load versus vertical displacement at the center.

Figure 5 shows the relationships between the applied axial load and vertical displacement at the center of the test specimens. From Figure 5, it can be found that the difference in the stiffness between the test specimens with and without PSPs was not significant. However, the strength and ductility of the test specimens with PSPs were considerably improved compared to the test result of the specimen without PSPs (P0_RC specimen). For P0_RC specimen, the ultimate strength, *P*_*u*_, was 2,105 kN and the applied load was suddenly dropped after reaching *P*_*u*_. On the other hand, for P5_3.2, P5_6, and P5H_3.2 specimens, two different peaks were observed. The applied loads were decreased after reaching the first peak. Then, the applied loads were continuously increased up to the second peak and a considerable additional deformation capacity was achieved. In this study, the applied loads corresponding to the first and the second peaks are defined as *P* ^*^ and *P* ^**^, respectively. The ultimate strength of the footing is then obtained as the maximum value of *P* ^*^ and *P* ^**^. *P* ^*^ and *P*_*u*_ of each of the specimens are shown in Table 1.

For P5_3.2 specimen, *P* ^*^ was larger than *P* ^**^. *P*_*u*_ of P5_3.2 specimen was 2,572 kN which is 22% larger than that of P0_RC specimen. In the case of P5_6 and P5H_3.2 specimens, *P* ^**^ was larger than *P* ^*^. *P*_*u*_ of P5_6 and P5H_3.2 specimens were 32% and 27% larger than that of P0_RC specimen. P7_3.2 specimen showed a single peak similarly with P0_RC specimen. *P*_*u*_ of P7_3.2 specimen was 12% larger than P0_RC specimen. It is interesting that P7_3.2 specimen showed the smallest increase in *P*_*u*_ among the test specimens with PSP, even if the size of PSP is larger than the other test specimens. The larger size of PSP did not provide a better performance of the footing. It can also be found that the thickness of PSP and holes in PSP also affect the behavior of the footing with PSP. By increasing the thickness of PSP, the ultimate strength was increased (refer to the results of P5_3.2 and P5_6 specimens). Further, approximately 10% increase in the ultimate strength was observed by introducing the punched holes in PSP (refer to the results of P5_3.2 and P5H_3.2 specimens), since the punched hole enhances the attachment between the steel and the concrete.

Figure 5 does not provide sufficient amount of information on the failure mode of the footing. Generally, the punching failure is divided into two different types [22, 23]. The first one is shear failure that occurs suddenly with a small displacement. This type of failure is frequently observed in the footing or slab with a large flexural reinforcement ratio. The second type of failure mode is the flexural failure. This failure takes place when the flexural reinforcement ratio is small, and the footing or slab is failed by the yielding of the reinforcing bar. The mixed punching shear-flexural failure is also possible. Thus, it is needed to evaluate the axial strain in the flexural reinforcing bar in order to classify the failure mode of the test specimens.

**Figure 6:**
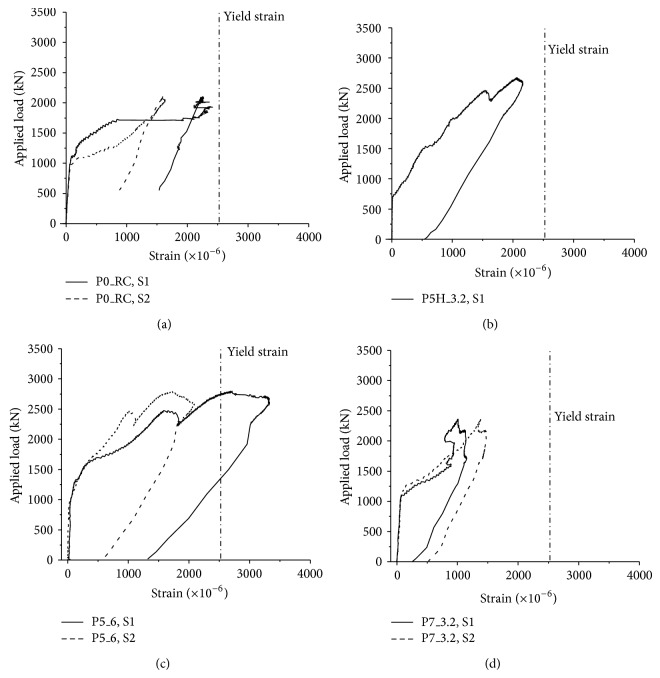
Applied load versus strain in the reinforcing bar: (a) P0_RC; (b) P5H_3.2; (c) P5_6; and (d) P7_3.2.

Figures 6(a)–6(d) show the axial strain data of flexural reinforcing bars of P0_RC, P5H_3.2, P5_6, and P7_3.2 specimens, respectively. The locations of S1 and S2 strain gauges are shown in Figure 3. It should be noted that the strain data of P5_3.2 specimen and S2 strain data of P5H_3.2 specimen were corrupted during the test, and these data are not shown in Figure 6. From Figure 6(a), it can be seen that the strain of flexural reinforcing bar of P0_RC specimen did not reach the yield strain, where the yield strain of the reinforcing bar is 2, 650 × 10^−6^. Further, the strain in the flexural reinforcing bar suddenly jumped without significant increasing of the applied load. Thus, it can be concluded that the P0_RC specimen was failed by punching shear failure. The axial strain of the flexural reinforcing bar of P7_3.2 specimen also did not achieve the yield strain, and the axial strain was relatively small compared to those of P5H_3.2 and P5_6 specimens, as shown in Figures 6(b)–6(d). From Figure 5, only one peak was observed for P7_3.2 and the applied load was considerably reduced after *P*_*u*_. Thus, it can be known that punching shear failure also occurred for P7_3.2 specimen, even if the large size of PSP (700 × 200 × 250) was installed into the footing.

Similar to the applied load-displacement relationship shown in Figure 5, axial strains were reduced near the first peak for P5H 3.2 and P5 6 specimens. Then, the axial strain is continuously increased by increasing the applied load. In particular, for the P5_6 specimen, the axial strain of the flexural reinforcing bar exceeded the yield strain, as shown in Figure 6(c), even though the yield plateau is not considerable. Then, ductility of P5_6 specimen was considerably increased. Thus, it can be found that PSPs could prevent the brittle punching shear failure by redistributing the applied load to the flexural reinforcing bars. P5H_3.2 specimen did not achieve the yield strain, and punching shear failure governed the strength of the footing. Also, the ductility of P5H_3.2 specimen was smaller than P5_6 specimen.

**Figure 7:**
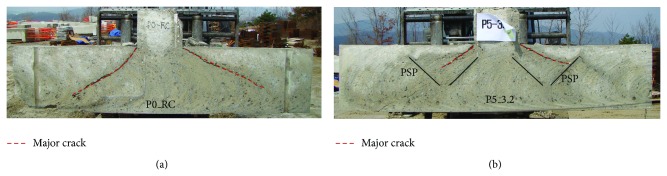
Crack pattern in the middle plane of the test specimen: (a) P0_RC and (b) P5_3.2.

**Figure 8:**
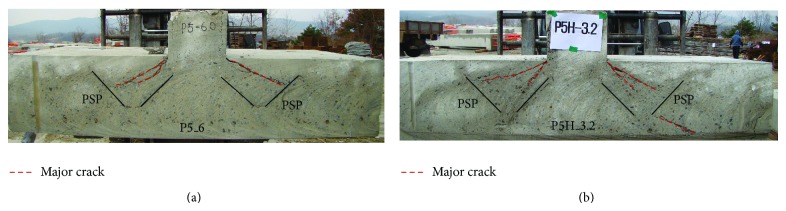
Crack pattern in the middle plane of the test specimen: (a) P5_6 and (b) P5H_3.2.

**Figure 9:**
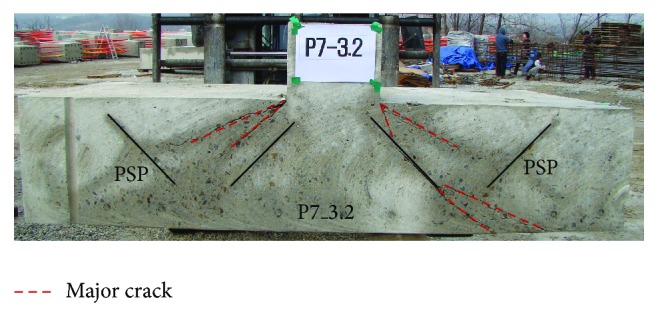
Crack pattern in the middle plane of P7_3.2 test specimen.

After the test, the specimens were cut to document the crack patterns. Figures 7–9 show the crack patterns of each test specimen. For P0_RC specimen, the cracks were developed in diagonal direction from the top to the bottom of the footing, as shown in Figure 7(a). This is a typical shear crack developed by punching shear. On the other hand, for P5_3.2, P5_6, and P5H_3.2 specimens, major cracks initiated at the interface between the column and the top surface of the footing were stopped approximately at the center of the exterior part of PSP, as shown in Figures 7(b), 8(a), and 8(b). As a result, shear cracks were isolated by PSP and the resistance was not significantly reduced. In the case of P7_3.2 specimen, it can be seen that the distance from the interface between the column and the top surface of the footing, where the shear crack initiated, to the exterior part of PSP is relatively far away compared to P5_3.2, P5_6, and P5H_3.2 specimens. Thus, the shear cracks were not effectively isolated. Taken as a whole, the following observations and conclusions were made from the experimental study.

- Introducing PSPs into the footing, the punching shear strength and ductility of the footing can be improved, since PSPs effectively isolate the shear crack and redistribute the applied load to the flexural reinforcing bars.
- By using proper PSPs, brittle punching shear failure can be prevented due to improved ductility.

However, the effectiveness of PSPs depends on their size. Further, the location of the PSP may affect the behavior of the footing. Thus, a series of parametric studies was undertaken to investigate the failure mechanism in depth and to investigate the effect of size, thickness, and location of PSP on the punching behavior of the footing by using nonlinear finite element analysis in the following sections.

## 3. Finite Element Analysis

### 3.1. Description of Finite Element Analysis Model

**Figure 10:**
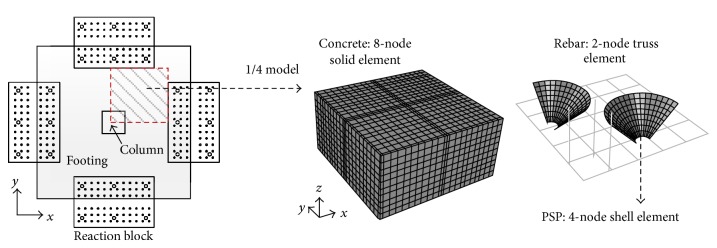
Typical finite element model for the test specimen.

Figure 10 shows the typical finite element model for the footing with PSP used in this study. The general purpose structural analysis program ABAQUS [24] was used. Quarter model was used for efficient modeling by taking advantage of the symmetry properties. The concrete footing was modeled using 8-node solid elements and PSP was modeled using 4-node shell elements. The 2-node truss element was used to model the reinforcing bars. PSP and the reinforcing bar were embedded into the concrete by using EMBEDED option in ABAQUS [24]. Thus, it was assumed that PSP and the reinforcing bar are perfectly bonded to the concrete. From the test, separation of interface between the PSP and the concrete was observed, and more accurate interface modeling may be needed to improve the finite element analysis model for the footing with PSPs. However, analysis results with full interaction interface between the PSP and the concrete show reasonable prediction of load-displacement relationship and crack patterns. Thus, to guarantee convergence of analysis, the perfectly bonded interface between the PSP and the concrete was used in this study.

**Figure 11:**
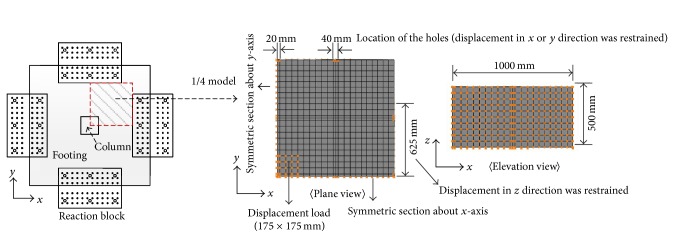
Loading and boundary condition of the finite element model.

Figure 11 represents the loading and the boundary conditions for the finite element models. Since the quarter model was used in this study, displacements in *x* and *y* direction were restrained for the left side and the bottom section, respectively, as shown in Figure 11. To simulate the boundary condition of the reaction block, displacements in *z* direction of the right and top edges were constrained, as shown in Figure 11 (only for 625 mm which is the half width of the reaction block. Refer to test setup shown in Figure 4). In addition, displacements in *x* and *y* direction in the location of anchor bar installed in the right and top edges were restrained, respectively. It is noted that the diameter of the anchor bar is 40 mm. Finally, the displacement loading in *z* direction was applied to simulate the load acting through the column.

**Figure 12:**
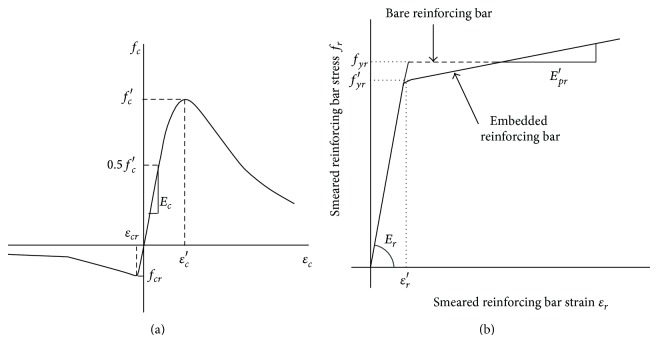
Material models: (a) concrete and (b) reinforcing bar.

Figures 12(a) and 12(b) show the uniaxial stress-strain relationship used for the concrete and the reinforcing bar in this study, respectively. Uniaxial compressive and tensile behavior of the plain concrete was modeled using expressions proposed by Saenz [25] and Hsu and Mo [26], respectively, where Young's modulus of the concrete, *E*_*c*_, was estimated as 4,700$\sqrt{f_{c}}$ (MPa) according to ACI design code [21]. It is also assumed that the stress-strain relationship of the concrete in compression is linear up to a stress of 0.5*f*_*c*_′ and the maximum compressive strength, *f*_*c*_′, is achieved when compressive strain is 0.003. Tensile stress-strain relationship is linear up to stress at cracking of concrete, *f*_*cr*_, and the softening relationship is given by the following equation [14]:

(1) $$\begin{array}{c} f_{c}=f_{cr}{\left(\frac{\varepsilon_{cr}}{\varepsilon_{c}}\right)}^{0.4}\;\text{when}\;\;\varepsilon_{c}\leq \varepsilon_{cr}. \end{array}$$

The tensile stress of the concrete, *f*_*cr*_, usually varies from 5% to 10% of *f*_*c*_′. In this study, a series of parametric studies was performed to evaluate the proper value of *f*_*cr*_, and *f*_*cr*_ was assumed to be 7.5% of *f*_*c*_′ based on the results of the parametric study. To simulate the inelastic behavior of the concrete under a general 3D stress state, the concrete damaged plasticity model incorporated in ABAQUS [24] was adopted. This model follows the nonassociated flow rule. Thus, plastic flow is governed by a flow potential function. The flow potential in concrete damaged plasticity model is a function of dilation angle, *ψ* [27]. The dilation angle of the concrete varies depending on the concrete properties. In this study, *ψ* of 31° was adopted for the analysis based on the results of Lee and Fenves [27].

The average stress-strain relationship of a reinforcing bar embedded in concrete is different from that of a bare reinforcing bar [26]. The primary difference is the lower effective yield stress of the reinforcing bar, *f*_*yr*_, as shown in Figure 12(b). In this study, the average stress-strain relationship of an embedded reinforcing bar proposed by Hsu and Mo [26] was adopted. The average stress-strain relationship of embedded reinforcing bar proposed by Hus and Mo [26] is given by

(2a) $$\begin{array}{c} f_{r}=E_{r}\varepsilon_{r}\;\text{when}\;\;f_{r}\leq f_{yr}^{\prime }, \end{array}$$

(2b) fr=(0.91−2B)fyr+(0.02+0.25B)Erεrwhen  fr>fyr′,

where

(3) $$\begin{array}{c} f_{yr}=\left(0.93-2B\right)f_{yr},\;B=\frac{1}{\rho }{\left(\frac{f_{cr}}{f_{yr}}\right)}^{1.5}. \end{array}$$

In (2a) and (2b), *E*_*r*_ is Young's modulus of the reinforcing bar where *E*_*r*_ is assumed as 200,000 MPa. *f*_*r*_ and *ε*_*r*_ are the stress and strain in the reinforcing bar, respectively. *f*_*yr*_′ is the reduced yield stress of embedded reinforcing bars. For PSPs, Young's modulus of the steel, *E*_*s*_, was approximated as 200,000 MPa and the yield stress was 400 MPa.

### 3.2. Verification of the Analysis Model and Failure Mechanism of Footing with PSP

**Figure 13:**
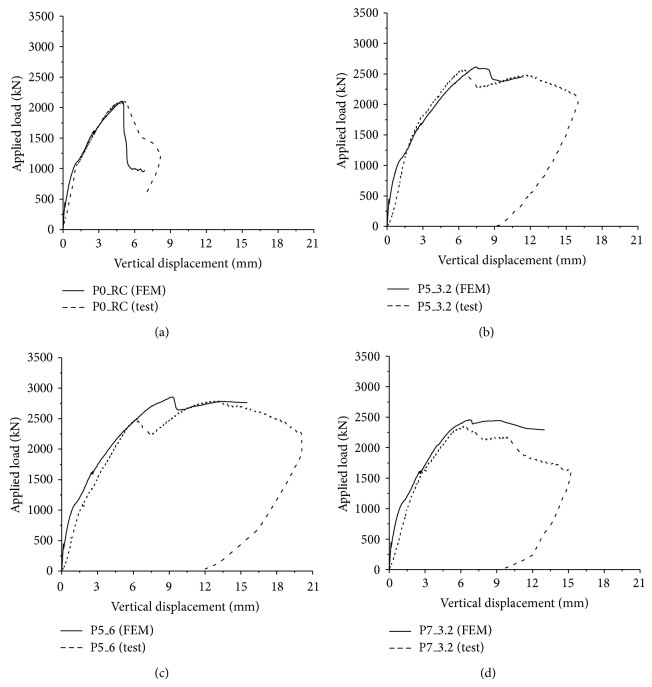
Comparison with test results: (a) P0_RC; (b) P5_3.2; (c) P5_6; and (d) P7_3.2.

All test specimens were modeled by using the methods described in the previous section except P5H_3.2 specimen. P5H_3.2 specimen was not simulated, since the PSPs are assumed to be perfectly bonded to the concrete in the analysis and the effect of punched holes on the PSP cannot be properly modeled in the analysis. Figures 13(a)–13(d) show the comparison of the results obtained from the analysis with those from tests for P0_RC, P5_3.2, P5_6, and P7_3.2 specimens, respectively. It can be seen that the analysis results agreed well with overall load-displacement relationships obtained from the test. The ultimate strengths of the analysis models were 2,103.7 kN, 2,615.5 kN, 2853.8 kN, and 2,456.2 kN for P0_RC, P5_3.2, P5_6, and P7_3.2 specimens, respectively. The maximum difference between the analysis results and the tests was approximately 4% for P7_3.2 specimen. The analysis provided good prediction for the ultimate strength of the test specimen.

**Figure 14:**
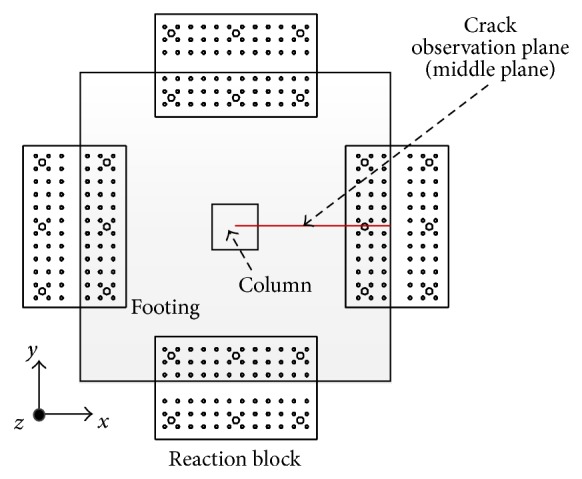
Location of crack observation plane for test specimens.

**Figure 15:**
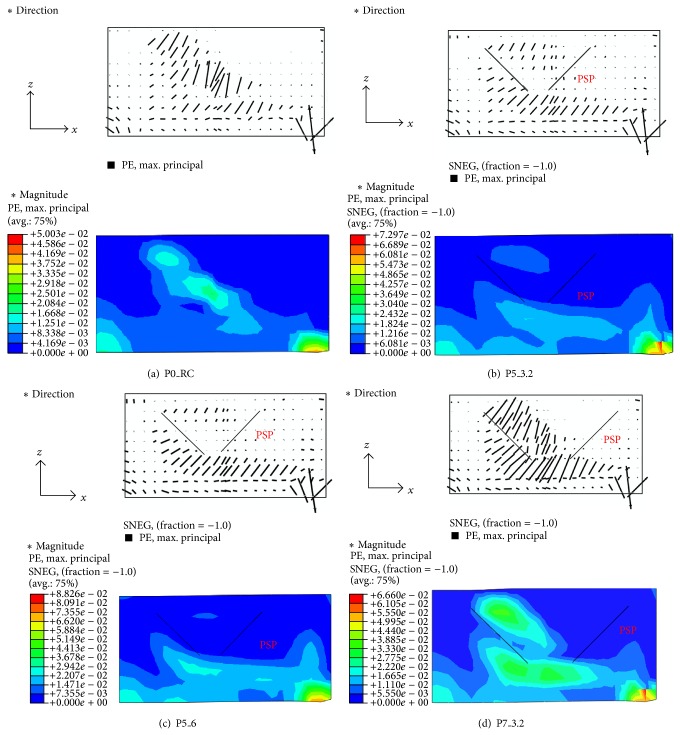
Crack patterns of the test specimens: (a) P0_RC; (b) P5_3.2; (c) P5_6; and (d) P7_3.2.

From the analysis results, crack patterns were evaluated at the middle plane shown in Figure 14. Figures 15(a)–15(d) represent the distribution of the maximum principle plastic tensile strain in the middle plane of the analysis models for P0_RC, P5_3.2, P5_6, and P7_3.2 specimens, respectively. In the concrete damaged plasticity model, it is assumed that crack initiates where the tensile equivalent plastic strain is greater than zero, and the maximum principal plastic strain is positive. The direction of the vector normal to the crack plane is assumed to be parallel to the direction of the maximum principal plastic strain [24]. Thus, the crack pattern can be evaluated by using the distribution of the maximum principle plastic tensile strain shown in Figure 15. From Figure 15, it can be seen that the major diagonal shear crack was developed and propagated to the bottom of the footing for P0_RC analysis model, as shown in Figure 15(a). This crack pattern was similar to the one obtained from the test (refer to Figure 7(a)).

On the other hand, for P5_3.2 and P5_6 analysis models, the diagonal shear cracks were effectively isolated by the PSP, as shown in Figures 15(b) and 15(c). Then, the flexural cracks below the PSPs were expended. As a result, the applied load redistributed to the flexural reinforcing bars. The diagonal shear crack of P5_6 analysis model was smaller than that of P5_3.2 analysis model. It resulted in greater ultimate strength of P5_6 analysis model than P5_3.2 model. In the case of P7_3.2 analysis model, similar to the test results shown in Figure 9, the shear cracks were not effectively isolated, and the relatively large diagonal shear crack was developed, as shown in Figure 15(d). As a result, the increase in the ultimate strength and ductility was limited. For all analyzed footings with PSPs, no significant cracks outside PSPs were observed.

Taken as a whole, it can be known that, as long as PSP can effectively isolate the diagonal shear cracks, the ultimate strength and ductility of the footing can be improved. The crack isolating ability of PSP depends on the size, thickness, and location of PSP. Thus, a series of parametric studies was performed to find the optimum size, thickness, and location of PSP. Details on the parametric study are presented in the following section.

## 4. Parametric Study

### 4.1. Description of Models for Parametric Study

**Figure 16:**
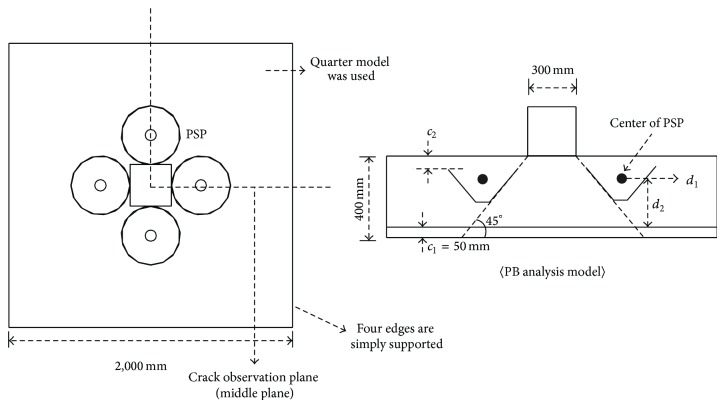
Dimensions of model for parametric study.

Figure 16 shows the dimensions of the models for parametric study. The concrete footing had a square shape with side length of 2,000 mm. The depth of the footing was 400 mm. The bottom flexural reinforcing bar consisted of SD500 D29 bars spaced at 150 mm, which results in reinforcement ratio of 1%. The nominal diameter of the SD500 D29 is 28.6 mm. The yield stress of SD500 D29 was assumed to be 530 MPa. The cover depth of the concrete, *c*_1_, was 50 mm. The compressive strength of the concrete was taken as 25 MPa. The compression was applied through 300 × 300 mm square area. Four PSPs were installed into the footing, as shown in Figure 16. The yield stress of the PSP was assumed to be 400 MPa. The quarter model was used for the analysis and the four bottom edges of the footing were assumed to be simply supported.

Table 2 shows the descriptions of the models for the parametric study. P_RC is the reference model for the conventional footing without PSPs. PB is the base model for the footing with PSPs where the top and the bottom diameter of the PSP (*d*_*t*_ and *d*_*b*_) were 350 mm and 50 mm, respectively. Thus, the PSP is inclined at a 45° angle. In this study, the angle of the slope of the PSP was limited to 45° for all test specimens and analysis models. The height of the PSP was 150 mm and the concrete cover depth for PSP, *c*_2_, was the same as that for the reinforcing bar (*c*_1_ = 50 mm). Thus, the summation of the concrete cover depth for PSP and the height of PSP is the same as half of the total depth of the footing.

The effect of the size, thickness, and location of PSP was investigated comparing the result of PB analysis model with those of PS, PT, and PL analysis model series, respectively. For PS_1 and PS_2 analysis models, the size of the PSPs was 450 × 150 × 150 and 650 × 350 × 150, respectively, while the thickness of PSP was the same at 3 mm. For PT_1 and PT_2 analysis models, the thickness of PSP was 1.5 and 6 mm, respectively, where the size of PSP was the same as that of PB model. In the case of PL_1 and PL_2 analysis models, the location of PSP was varied by relocating the center of the PSP. *d*_1_ and *d*_2_ shown in Figure 16 are the horizontal and the vertical distance from the original center of PSP to the new center location of the PSP. The values of *d*_1_ and *d*_2_ for PL_1 and PL_2 analysis models are shown in Table 2.

### 4.2. Results of Parametric Study and Design Recommendation

**Figure 17:**
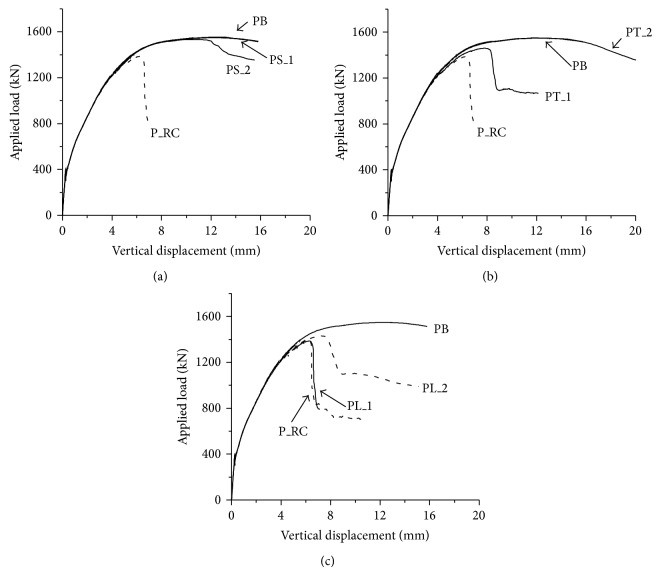
Results of parametric study (load-displacement relationship): (a) effect of the size of PSP; (b) effect of thickness of the PSP; and (c) effect of the location of PSP.

**Figure 18:**
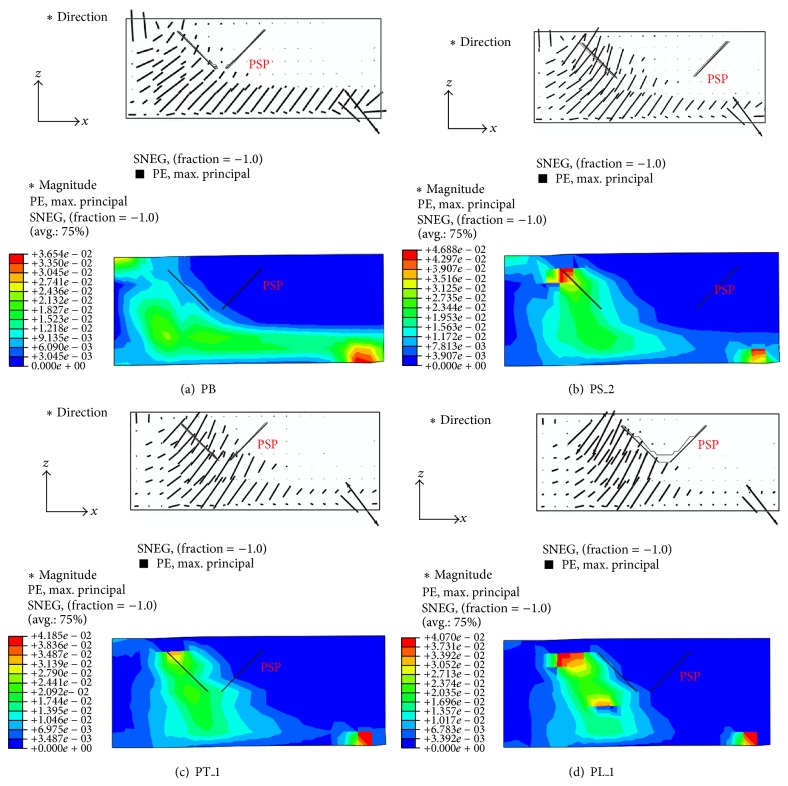
Cracking patterns of analysis models: (a) PB; (b) PS_2; (c) PT_1; and (d) PL_1.

Figures 17 and 18 show the applied load-vertical displacement relationships and crack patterns of the analysis models, respectively. From Figure 17(a), it can be seen that the strength and ductility of the footing with PSPs were considerably increased by comparing with the analysis result of P_RC model. PB model showed approximately 12% larger strength than P_RC model.

It is noted that the deformation capacity of PS_2 model decreased comparing with PB and PS_1 models, as shown in Figure 17(a). This is due to the large size of the PSP, which makes it unsuitable to prevent the diagonal shear crack. From the crack patters of PB and PS_2 analysis models shown in Figures 18(a) and 18(b), it can be seen that considerable diagonal shear crack was developed for PS_2 analysis model, while the major crack in PB analysis model was developed by the flexure.

In the parametric study, the height of the PSP was fixed. Thus, the size of PSP varies depending on the ratio between the top and the bottom diameter of PSP, *d*_*t*_/*d*_*b*_. Smaller value of *d*_*t*_/*d*_*b*_ results in larger size of the PSP. As a result, *d*_*t*_/*d*_*b*_ should be large enough to effectively isolate the diagonal shear crack and to increase the strength and ductility of the footing. Based on the results of tests and parametric study, the shear crack was effectively prevented for P5_3.2, P5_6_P5H_3.2, PB, and PT_2 test specimens or analysis models. For these models, *d*_*t*_/*d*_*b*_ varied from 5 to 7, and these values are recommended.

The effect of the thickness of PSP on the behavior of the footing can be evaluated from Figure 17(b). The thickness of PSP was normalized by the height of PSP as *h*/*t*. Smaller *h*/*t* represents a larger thickness of PSP. For PB, PT_1, and PT_2 analysis models, *h*/*t* were 50, 100, and 25, respectively. When the thickness of PSP was small, such as PT_1 analysis model (*h*/*t* = 100), the significant diagonal shear crack was observed, as shown in Figure 18(c), and the increase in the strength and ductility was limited, as shown in Figure 17(b). For PB and PT_2 analysis models, where *h*/*t* is smaller than 50, the analysis results were almost identical to each other, as shown in Figure 17(b). Based on the test and analysis results, the PSP shows a good performance when *h*/*t* is smaller than 62.5, and this value is recommended for the design purpose.

Figure 17(c) represents the effect of the location of PSP on the behavior of the footing. For both PL_1 and PL_2 analysis models, the applied load-vertical displacement relationships were similar compared to that of the footing without PSP (P_RC), as shown in Figure 17(c). In particular, for PL_1 analysis model, the behavior is almost identical to that of P_RC analysis model. The crack pattern of PL_1 analysis model is presented in Figure 18(d). It can be seen that PSP is located outside the compression strut and the diagonal shear crack governs the behavior of the footing. In the case of PL_2 analysis model, the location of PSP was in the zone of the compressive strut. However, PSP was placed just above the bottom flexural reinforcing bars and the distance from the PSP to the interface between the column and the top surface of the footing is relatively far away compared to other analysis models. Thus, it can be concluded that PSP should be placed in the zone of the compressive strut and as close to the top surface of the footing as possible to take the benefits of the PSP.

Finally, the following initial design recommendations for the footing with PSPs were suggested.

- *c*_2_ + *h* should not exceed or be similar to the half of the depth of the footing, where *c*_2_ and *h* are the concrete cover depth for PSP and height of the PSP, respectively.
- *d*_*t*_/*d*_*b*_ should be ranged from 5 to 7. *h*/*t* should be smaller than 62.5. But, when *h*/*t* = 25, there is no increase in the strength and deformation capacity from the analysis results.
- To take the benefit of PSP, PSP should be placed at the zone of the compressive strut.

### 4.3. Comparison with ACI Design Code

According to ACI [21], the punching shear strength of the slab or the footing subjected to a square column can be determined as

(4) $$\begin{array}{c} 0.33\sqrt{f_{c}^{\prime }}\lambda b_{0}d\;\left(\text{in}\;\text{MPa}\right), \end{array}$$

where *b*_0_ is the control perimeter and it is 0.5*d* from the loaded area. *d* is the effective depth of the slab or the footing. *λ* in (4) is a modification factor to take into account the effect of lightweight concrete. For the normal weight concrete, *λ* is equal to 1.

The ultimate strength of the test specimens and analysis results that meet the proposed initial design recommendations were compared with (4) in this section. The comparison results are shown in Table 3. From Table 3, it can be found that (4) agrees well with the punching shear strength of the footing without PSP. The average discrepancy was 2%. For the footings with PSPs that meet the proposed design recommendations, ACI design code [21] underestimates the ultimate strength of the footing by 14%. The strength of the footing with PSPs was approximately 16% higher than that of the footing without PSPs.

## 5. Conclusions and Further Study

This study presents a new method to improve the strength and ductility of the footing by inserting the PSPs into the footing. The validation and effectiveness of the proposed method were verified by a series of tests and nonlinear finite element analysis. From the test results, it can be found that the strength and ductility of the footing were considerably increased by using the PSPs, since the diagonal shear cracks can be effectively isolated by PSPs. Then, the applied load redistributed to the flexural reinforcing bars.

The nonlinear finite element analysis model was constructed and successfully verified by comparing with the test results. Then, a series of parametric studies was conducted to investigate the effect of the size, thickness, and location of PSP on the behavior of the footing with PSPs. From the results of parametric study, it can be found that PSPs should be placed in the zone of the compressive strut to take the benefit of PSP. Further, the analysis results show that increase in the size and thickness of the PSP does not always guarantee a better strength and ductility of the footing with PSPs. Based on the results of the test and the parametric study, initial design recommendations for the footing with PSPs were proposed.

Finally, the strengths obtained from the test and parametric study that meet the proposed design recommendations were compared with ACI design equation. ACI design equation underestimates the strength of the footing with PSPs by approximately 14%. To examine the efficiency of the proposed footing with PSPs, a comparison to a footing with conventional punching shear reinforcements is necessary. Also, more systematic comparison with current design codes and development of mechanical model for the footing with PSPs that explain the enhancement of the punching shear performance is needed in the future study.

## Figures and Tables

**Table 1:** Description of test specimens and test results.

Name	PSP (*d*_*t*_ × *d*_*b*_ × *h*) (mm)	*t* (mm)	*d*_*t*_/*d*_*b*_	*h*/*t*	Test parameter	*P* ^*^ (kN)	*P*_*u*_ (kN)
P0_RC	None	None	None	None	Reference model	2,105	2,105
P5_3.2	500 × 100 × 200	3.2	5	62.5	Effect of PSP	2,572	2,572
P5_6	500 × 100 × 200	6	5	33.3	Effect of thickness of PSP	2,433	2,796
P5H_3.2	500 × 100 × 200 (with 6 holes)	3.2	5	62.5	Effect of holes in PSP	2,454	2,675
P7_3.2	700 × 200 × 250	3.2	3.5	78.1	Effect of size of PSP	2,361	2,361

**Table 2:** Models for parametric study.

Name	PSP (*d*_*t*_ × *d*_*b*_ × *h*) (mm)	*t* (mm)	*d*_*t*_/*d*_*b*_	*h*/*t*	*d*_1_ (mm)	*d*_2_ (mm)	Study parameter
P_RC	None	None	None	None	None	None	Reference model
PB	350 × 50 × 150	3	7	50	0	0	Reference model and effect of PSP
PS_1	450 × 150 × 150	3	3	50	0	0	Effect of size of PSP
PS_2	650 × 350 × 150	3	1.9	50	0	0	Effect of size of PSP
PT_1	350 × 50 × 150	1.5	7	100	0	0	Effect of thickness of PSP
PT_2	350 × 50 × 150	6	7	25	0	0	Effect of thickness of PSP
PL_1	350 × 50 × 150	3	7	50	150	0	Effect of location of PSP
PL_2	350 × 50 × 150	3	7	50	100	100	Effect of location of PSP

**Table:** (a) The foundation without PSP

Name	*P*_*u*,test or FEM_/*P*_*u*,ACI_	Remark
Po_RC	1.03	Test
P_RC	0.92	FEM

Average	0.98	

**Table:** (b) The foundation with PSP that meets the design recommendation proposed in this study

Name	*P*_*u*,test or FEM_/*P*_*u*,ACI_	Remark
P5_3.2	1.26	Test
P5_6	1.19	Test
P5H_3.2	1.20	Test
PB	1.03	FEM
PT_2	1.03	FEM

Average	1.14	—
